# Research progress on molecular mechanism of liver metastasis of gastric cancer and treatment with traditional Chinese medicine

**DOI:** 10.7150/jca.105223

**Published:** 2025-03-03

**Authors:** Caiyue Liu, Zheng Li, Fane Cheng, Weiqiang Li, Tingting Li

**Affiliations:** 1 Ningxia Medical University School of Traditional Chinese Medicine, Yinchuan, P. R. China.; 2 The First Clinical Medical College of Beijing University of Traditional Chinese Medicine, Beijing, P. R. China.; 3 Key Laboratory of Ningxia Minority Medicine Modernization, Ministry of Education (Ningxia Medical University), Yinchuan, P. R. China.; 4 Ningxia medical university affiliated traditional Chinese medicine hospital, Yinchuan, P. R. China.

**Keywords:** gastric cancer, liver metastasis, traditional chinese medicine, tumor

## Abstract

Gastric cancer liver metastasis (GCLM) refers to the process of cancer cells from the stomach spreading to the liver, which is an important sign of the deterioration of gastric cancer (GC) and has a profound influence on the treatment and prognosis of patients. Once GC has liver metastasis, the treatment becomes more complex and challenging, which seriously affects the survival rate of patients with GC. Therefore, studying the mechanism and treatment of GCLM is extremely necessary. At present, the continuous research on GCLM has revealed that the mechanism of its occurrence and development involves the comprehensive effect of multiple targets and links. Traditional Chinese medicine (TCM) has the advantages of wide sources, excellent efficacy, and small toxicity and side effects, which have become the focus of current antitumor research. TCM, Chinese medicine monomers, or TCM compounds can inhibit the growth and metastasis of GC. In recent years, Chinese medicine has made substantial achievements in experimental research on the intervention of GCLM. This article reviews the progress of its intervention mechanism.

## Introduction

Gastric cancer (GC) is one of the most common malignant tumors in clinics. According to the international cancer research institute GLOBOCAN, gastric cancer ranks fifth in the global cancer incidence rate in 2020, and is the third leading cause of cancer-related deaths, with approximately 769,000 deaths^1^. The incidence of GC has evident regional characteristics, and it is usually more common in parts of East and Eastern Europe and South America. The incidence and mortality of GC are related to many factors such as environmental factors, dietary habits, and genetic factors. The main risk factors for helicobacter pylori infection are smoking and high salt intake^2^,^3^. Although the prevention and treatment of GC have improved in recent years, because the early symptoms of stomach cancer are hidden, and the specific markers for early diagnosis of most of the patients are lacking or found in the middle-late stages of GC invasion and metastasis, the survival rate of patients is seriously affected. In the past two decades, the incidence of GC metastasis has increased substantially^4^. Distant lymph node metastasis (56%), liver metastasis (53%), and peritoneal metastasis (51%) are the most common metastasis types of GC, whereas gastrointestinal gastric adenocarcinoma is more likely to metastasize to the liver^5^. Gastric cancer liver metastasis (GCLM) has an overall incidence of about 9.9% to 18.7%. The prognosis of GCLM is poor, and the five-year survival rate of patients is less than 20%^6^. Therefore, GCLM is a clinical problem worthy of attention.

Although some progress has been made in the treatment of GCLM in recent years, no standardized chemotherapy regimen has been established internationally due to the different tolerances of patients. Currently, the main treatment for GCLM is surgery, radiotherapy, and systemic therapy (chemotherapy, targeted therapy, and immunotherapy)^7^. Surgical resection is the method of choice for the radical treatment of GCLM. However, unlike colorectal liver metastasis (disease that manifests only in the liver and is amenable to hepatic resection), GCLM tends to be multifocal or double-barreled and is often accompanied by metastases to the peritoneum or lymph nodes; hence, it limits the applicability of surgery and entails higher postoperative complications and risks^8^. According to the National Comprehensive Cancer Network, liver metastasis is classified as stage IVb disease, and systemic chemotherapy is the main treatment for GCLM, including oxaliplatin and fluorouracil^9^. However, systemic chemotherapy has greater side effects and may affect patients' quality of life. In recent years, studies have shown that hepatectomy and gastrectomy plus chemotherapy and radiofrequency ablation and gastrectomy plus chemotherapy are the most effective options for the treatment of GCLM^10^. Such interventional, surgical treatments can also lead to toxic side effects and cause greater physical and psychological harm to patients.

Traditional Chinese medicine (TCM) is a kind of natural medicine, and many TCMs and their components have shown great potential in modern drug research and development. Chinese medicines contain numerous plant, animal, and mineral components, and offer a rich diversity of chemical structures, which provide a wide range of lead compounds for drug research and development. Moreover, Chinese medicines have few side effects and harm to the human body. Chinese medicines can stimulate the body's immune system and enhance the patient's own anticancer ability, helping control tumor growth and metastasis^11^. Their overall regulatory effects through multiple components, targets, and pathways allow them to play a variety of roles in treatment, and comprehensively improve patient symptoms and prolong survival. For example, baicalein (BAI), a Chinese herbal component derived from the Chinese medicine scutellaria baicalensis can exert antitumor effects by inhibiting proliferation and angiogenesis as well as invasion and metastasis, and regulating cellular signal transduction pathways. In recent years, many clinical and research studies have confirmed that TCM has a good effect on antitumor and inhibition of metastasis, and plays an increasingly important role in the treatment of cancer-related diseases^12^. Therefore, studying the mechanism of the occurrence and metastasis of liver metastasis of GC by TCM has great clinical value. In this study, the mechanism of inhibition of liver metastasis of GC by TCM and the progress of research on TCM treatment of GC are summarized to provide a systematic theoretical basis and practical guidance for early diagnosis, optimization of treatment strategies, and improvement of patient prognosis.

## Molecular mechanism of liver metastasis in gastric cancer

**Figure 1:**
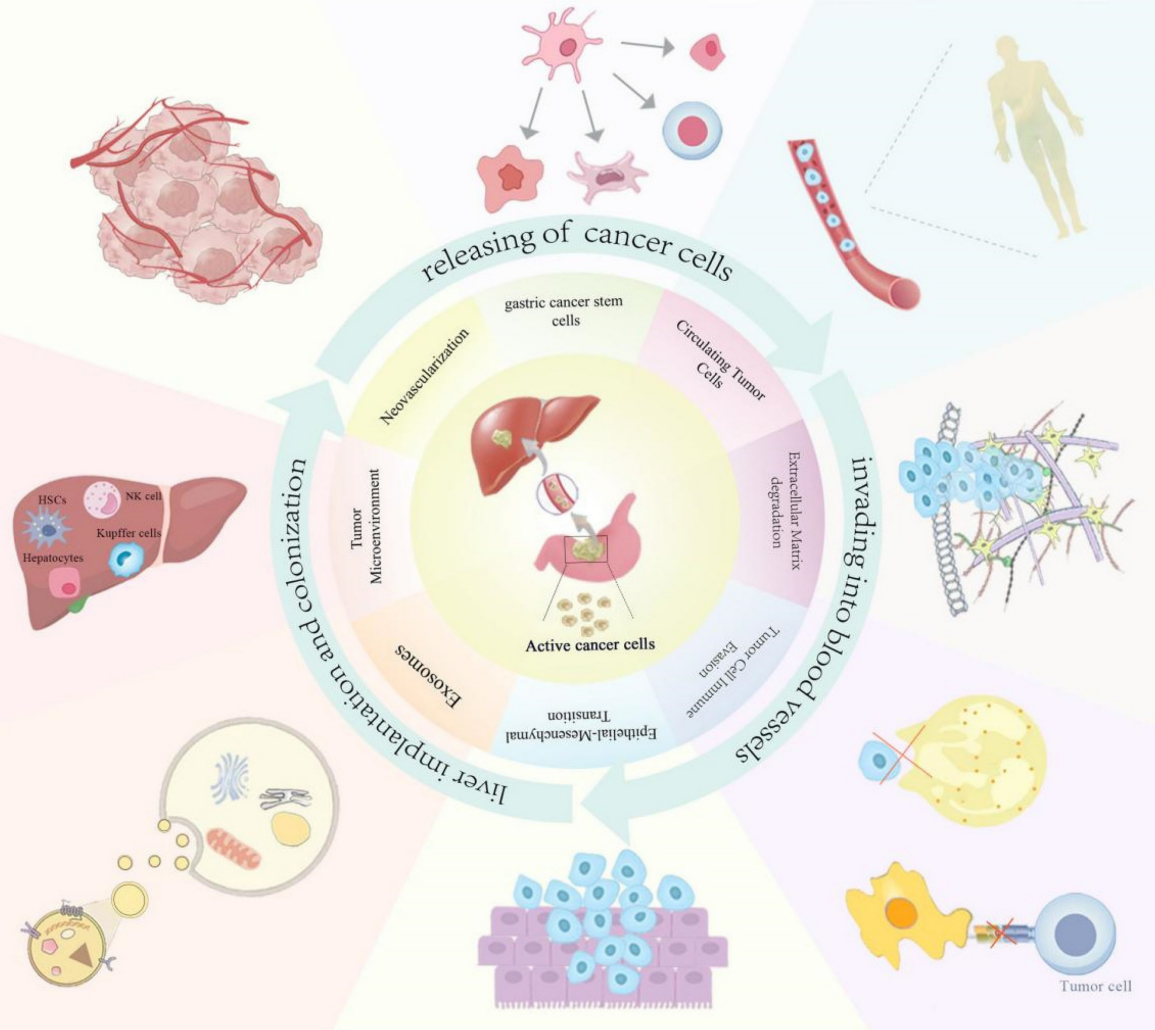
The main mechanisms of GCLM.

GCLM refers to the metastasis of GC cells to the liver, forming a metastatic tumor of the liver, which occurs depending on the malignant transformation of GC cells and the interaction with the liver microenvironment. The modes of GC metastasis mainly include blood metastasis, lymphatic metastasis, direct invasion, and implantation metastasis. The liver is supplied with blood by the portal vein and the hepatic artery, of which 1/4 comes from the hepatic artery, and the remaining 3/4 comes from the portal vein, which is a collection of veins from the stomach, intestines, spleen, and other organs, and which transfers various nutrients and harmful substances from the digestive tract to the liver^13^. Therefore, this blood flow characteristic of the liver makes hematological metastasis the main mode of realization of GCLM. GC cells metastasize through the blood system. This process can be divided into three steps: First, cancer cells with high infiltration and metastatic ability separate from the primary tumor, invade the blood vessels, become circulating tumor cells (CTCs), enter the portal vein, and finally implant and proliferate in the target organ liver to form metastatic lesions^14^. The mechanism of this complex biological process involves the interactions of multiple factors including tumor cell invasion, blood circulation, tumor microenvironment changes, genetic and molecular changes, and the role of the immune system. This article summarizes the current molecular mechanisms of GCLM in terms of epithelial-mesenchymal transition (EMT), degradation of the extracellular matrix (ECM), tumor stem cells, exosomes, circulating tumor cells, tumor microenvironment, angiogenesis, and immune escape (Figure 1). Understanding the complex mechanisms of GCLM is essential for developing effective preventive and therapeutic approaches.

### EMT process

**Figure 2:**
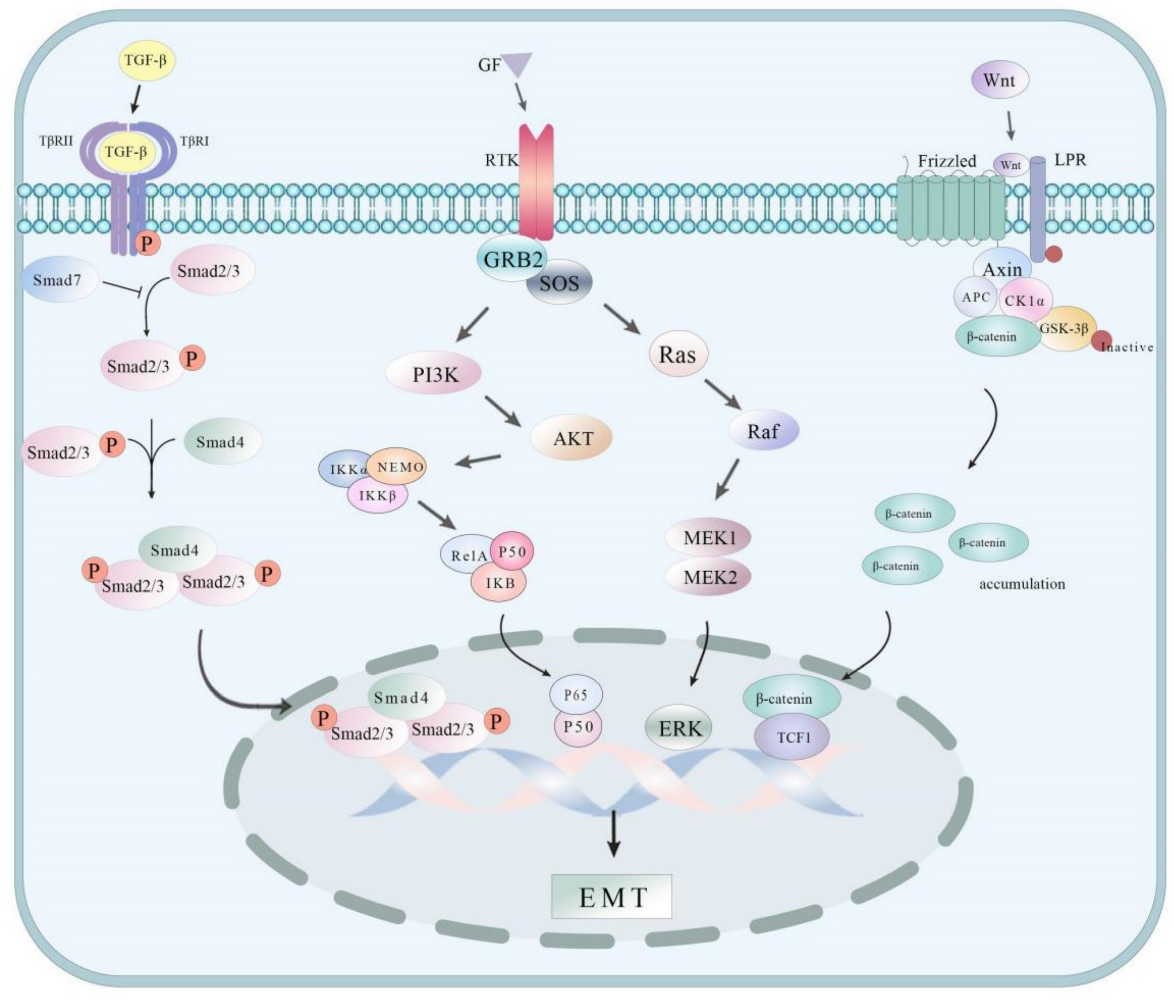
Some mechanism pathways of EMT processes.

EMT refers to the process of epithelial cells losing their cell polarity and cell-cell junctions and transforming into more mobile, invasive mesenchymal cells, which enable cells to escape from the primary tumor and invade the surrounding tissues or blood vessels, thereby promoting the distant metastasis of tumors. Understanding some of the pathways associated with EMT can help better understand the molecular mechanism of its occurrence and development (Figure 2). In GCLM, EMT plays an important role, which is usually closely associated with the downregulation of epithelial markers (such as E-cadherin) and the upregulation of mesenchymal markers (such as N-cadherin and Vimentin). Receptor-like tyrosine kinase (RTK) is highly correlated with hepatic metastasis and EMT progression in GC tumors. This study revealed *in vitro* (tumor cell samples collected from 250 patients) and *in vivo* (using a mouse xenograft model) that RTK is positively correlated with GC and spreads to the liver tumor; the mechanism may be caused by the interaction with Wnt5a in the noncanonical Wnt signaling pathway^15^. Moreover, the study found that miR-130a-3p can directly target and inhibit TBL1XR1, which considerably increases the expression of E-cadherin in MGC-803 cells, decreases the expression of N-cadherin in MGC-2 cells and MGC-803 cells, and inhibits EMT, thus suppressing the motility and invasiveness of cancer cells and preventing tumor metastasis^16^. A study by Li *et al.*^17^ showed that Sema3C is highly expressed in GC cells with highly metastatic characteristics. CCK 8, Transwell invasion, and migration experiments show that Sema3C knock low-energy substantial inhibition of GC cell proliferation, invasion, and migration; Western blot and immunohistochemistry experiment found silence Sema3C can increase the expression of E-cadherin, the expression of Vimentin. It mediates EMT to promote GC formation and liver metastasis. Snail and Slug play key roles in the development of cancer, especially in EMT and tumor metastasis. miR-33a blocks the activation of the Snail/Slug signaling pathway by targeting and inhibiting the expression of SNAI2, inhibiting the proliferation, invasion, and metastasis of GC cells^18^. Xie *et al.*^7^ found in the research on miR-582 and the correlation of liver metastasis that miR-582 in GC cells and tissues have a considerably high expression. Further study on the mechanism of miR-582 overexpression and GC cell invasion and metastasis found that miR-582 interacts with FOXO3 to activate PI3K/Akt/Snail pathway and promote GC cell invasion and migration. In addition, the study by Zhou *et al.*^19^ suggested that TBL1XR1, as a regulator closely related to tumorigenesis, could promote GC tumor cell proliferation, migration, invasion, and EMT by activating the β-catenin/MMP7/EGFR/ERK signaling pathway. Chen *et al.*^20^ found that the high expression of Alpha B-crystallin (CRYAB) is highly correlated with cancer metastasis, and the mechanism is that the upregulation of CRYAB expression can promote the migration and invasion of GC cells by activating EMT mediated by the NF-κB signaling pathway.

### ECM degradation

ECM is the living environment of cells, which is composed of a variety of proteins such as collagen, elastin, and laminin. ECM can help cells maintain their morphology, ensure the normal performance of physiological functions, and participate in the signal transduction between cells. The degradation of ECM in liver metastatic GC is more than a link, involving a variety of molecular and cellular interactions of a process. Some enzymes can rupture the structure of the extracellular matrix, such as matrix metalloproteinases (MMPs) secreted by GC cells that break down collagen and fibronectin in the ECM^21^, providing a pathway for GC cells to invade and migrate. One of the functions of disintegrin-metalloproteinases (ADAM8) is the ability to influence multiple cytokine functions involved in ECM degradation. ADAM8 hydrolyzes a variety of protein components in the ECM to help tumor cells enter the blood circulation. ADAM8 can also release growth factors acting on tumor cells and promote the proliferation and migration of tumor cells^22^. The degradation of the ECM can also change the tumor microenvironment, such as adjusting extracellular pH, releasing cytokines and growth factors, and creating more favorable conditions for tumor growth and metastasis. For example, MMP-9 can release vascular endothelial growth factor (VEGF) to support the creation of new blood vessels and maintain nutrition for tumor cells^23^. In addition, changes in ECM components may hide tumor cells and make them difficult to recognize and eliminate by immune cells, so the degradation of ECM can affect the immune surveillance of tumor cells in the liver^22^.

### Gastric cancer stem cells

Tumor stem cells are a kind of tumor cells with stem cell characteristics, which play a key role in the occurrence, development, recurrence, and metastasis of tumors^24^. GC stem cells have the ability of unlimited self-renewal, differentiation, and tumor regeneration, which makes them play a key role in the drug resistance, recurrence, and metastasis of GC^25^. These cells have a high degree of invasion and migration ability. They can secrete some proangiogenic factors, chemokines, and other substances; activate alternative transcription factors; initiate downstream signaling pathways; promote downstream cells to produce micro angiogenesis; and increase the blood supply of tumor cells and enhance their metastatic ability^26^,^27^. In addition, GC stem cells can induce a local inflammatory response by secreting some proinflammatory cytokines, destroy the body's defense system, reduce antitumor immunity, and promote the migration and invasion of tumor cells^28^. In conclusion, GC stem cells play a key role in GCLM, and an in-depth understanding of the mechanism is important for the development of new therapeutic strategies.

### Exosomes

Exosomes are small vesicles secreted by cells containing a variety of biomolecules such as proteins, RNA, and DNA fragments, which enable intercellular information exchange and material transport^29^. In recent years, many studies have found that exosomes of tumor cells play an important role in cancer formation and progression by mediating the remodeling of the tumor microenvironment, promoting angiogenesis, aiding cancer cell immune escape, inducing EMT, and initiating or inhibiting various signaling pathways-induced liver metastasis before the formation of ecological niche^7^. Qiu *et al.*^30^ showed that GC-derived exosomal miR-519a-3p activates the MAPK/ERK pathway by targeting DUSP2, a member of the dual-specificity protein phosphatase subfamily, to induce the M2-like polarization of macrophages, thus triggering angiogenesis and prehepatic metastasis niche formation to promote liver metastasis. Zhang *et al.*^31^ found that exosomes derived from GC cells can transfer EGFR to the hepatocyte membrane, thereby activating hepatocyte growth factor in the liver by inhibiting miR-26a/b in hepatocytes and promoting the adhesion and proliferation of GC cells in the liver. This study provides data validation for the “seed and soil” hypothesis mentioned next, reveals a new mechanism of liver metastasis, and provides effective strategies to guide clinical treatment. However, the deficiency of this study is that when conducting experiments to verify the mechanism of GCLM by EGFR in exosomes, the silencing of EGFR may lead to changes in exosome content, which in turn may affect the experimental data. In addition, Feng *et al.*^32^ found that exosomal miR-196a-1 regulates SFRP1, binds to the 3' untranslated region of SFRP1 in cancer cells, and promotes GC invasion and liver metastasis by using luciferase reporter assay, bioinformatic analysis, Western blot, and other experimental methods.

### CTCs

CTCs are tumor cells that shed from solid tumor lesions (primary tumor and metastatic tumor) and enter the peripheral blood^33^. These cells are shed from solid tumor lesions, and the majority of these cells undergo apoptosis or are phagocytosed upon entry into the peripheral bloodstream, but a few escape and develop into metastatic lesions. In the new environment of the liver, CTCs need to adapt to the changes in the liver microenvironment and interact with a variety of cell types in the liver, including hepatocytes, hepatic sinusoidal endothelial cells, hepatic stellate cells, and Kupffer cells (KCs). These cells can affect the liver microenvironment and help engraftment of CTC in the liver and the formation of metastases^34^. This process plays a key role in the metastasis of GC cells to the liver. Some studies have found that CTCs can work with the EMT, another mechanism of GCLM, to achieve higher mobility and invasiveness, so that they can successfully invade blood vessels from the primary tumor and become circulating tumor cells^35^. In addition, studies on CTCs in the diagnosis and prognosis prediction of GC have made some progress. For example, several studies have found that the number of CTCs in the peripheral blood of GC patients is associated with disease severity and prognosis^36^,^37^. A meta-analysis^38^ showed that patients with GC with detectable CTCs have shorter recurrence-free survival and overall survival. Recent studies have shown that different subclasses of CTCS play a key role in the mechanism of drug resistance in GCLM. In particular, triploid small cell CTC (SCTCtri) and polyploid large cell CTC (LCTCmulti) in patients with GC are closely associated with GCLM. Specifically, pretreatment small-cell CTCS has a high association with liver metastasis in GC. Pretreatment patients containing ≥3 SCTCtri or ≥6 LCTCmulti have a poorer prognosis and shorter median progression-free survival (mPFS). Pretreatment patients containing ≥6 LCTCmulti also have substantially shorter median overall survival^39^. These CTC subclasses have important clinical importance in the prognosis, efficacy prediction, recurrence, and metastasis of GC, and emphasize the importance of CTC detection in the management of GC patients with liver metastasis.

### Tumor microenvironment

GCLM involves in GC cells and the interaction between hepatic microenvironment. The liver microenvironment includes various cells, cytokines, extracellular matrix, and other components in the liver, which together constitute a complex ecosystem^40^, and have an important effect on the growth, diffusion, and colonization of GC cells. “Seed and soil” theory proposed by Step Paget shows that tumor metastasis is tenacious, not random, and the interaction between tumor cells as the “seed” and the “soil” that provides a suitable living environment promotes tumor metastasis^34^,^40^. Liver tissue mainly includes liver cells, liver sinusoidal endothelial cells (LSEC), hepatic stellate cells (HSC), KCs, and other types. These intrinsic hepatic cells interact with tumor-secreted factors to trigger inflammatory responses, promote neovascularization, and aid in immune tolerance, among other responses that contribute to the GCLM^34^. Although LSEC initially prevents tumor cells from entering the liver by inducing cell death via the Fas-FasL pathway, with continuous stimulation of tumor cells, LSEC instead promotes tumor cell colonization in the liver by inducing angiogenesis, increases the adhesion of tumor cells to the endothelium, and promotes the immunosuppression of the tumor cells. HSCS is also important for liver metastasis. Activated HSCS facilitates ECM fibrosis and then the formation of liver metastases by inhibiting immune response and promoting angiogenesis, tumor cell proliferation, and metastasis. Xie *et al.*^41^ found in their study on the relationship between tumor-secreted proteins and the GCLM mechanism that GC-derived lipopolysaccharide-binding protein (LBP) is closely related to the occurrence of the GCLM mechanism. Internal bioluminescence imaging found that the overexpression of LBP in GC cells aggravates the progression of LM after intrasplenic injection. It can also activate HSC by stimulating intrahepatic macrophages to secrete TGF-β1, induce the formation of a pre-metastatic niche in liver fibrosis, and promote the occurrence of GCLM. Again, KC cells can phagocytose tumor cells to induce their apoptosis, but they indicate the presence of carcinoembryonic antigen receptors, and this activation is conducive to the formation of a survival environment for tumor cells^42^. In conclusion, the effect of the liver microenvironment on GCLM is realized through various components and signaling molecules.

### New blood vessel formation

Vascular metastasis is the main mode of GCLM. To grow and spread, tumors need to induce new angiogenesis to provide oxygen and nutrients. The formation of new blood vessels not only provides nutrients to the tumor but may also offer a route for tumor cells to enter the circulatory system^43^. After the tumor cells spread to the distant organization, a new blood supply needs to be established to support the growth of metastases and to help tumor cells in the liver build new blood vessel networks, thus promoting tumor invasion and metastasis. One study^44^ showed that tumor size, Borrmann type, tumor differentiation and invasion, lymph node metastasis, and the expression of vascular endothelial growth factors VEGF and VEGFR2 are remarkably correlated with micro vessel density in tumor tissue. Past studies have shown that VEGF can be used as independent prognostic index of the recurrence of patients with GC with liver and sensitive index; hence, VEGF plays an important role in GCLM^45^. Elevated plasma angiopoietin-2 (Ang-2) levels in patients with GC are closely related to the frequency of liver metastasis, and Ang-2 expression is induced by VEGF^46^. Angiogenesis affects not only the physical migration of tumor cells but also the tumor microenvironment. The instability of new blood vessels may lead to inflammation and immunosuppression in the tumor microenvironment, thereby favoring tumor cell survival and metastasis formation^47^,^48^. Moreover, angiogenesis is associated with tumor treatment resistance, and the abnormal structure and function of neovasculature may lead to insufficient drug delivery, rendering the tumor cells resistant to chemotherapy and radiotherapy^49^,^50^.

### Immune escape

Immune escape refers to the survival of tumor cells through a series of mechanisms to avoid recognition and clearance by the host's immune system^51^. In GCLM, this escape mechanism enables tumor cells to survive, grow, and spread in the new liver microenvironment. One report stated the mechanisms by which tumor cells evade immunity by regulating antigen expression, interfering with antigen processing and presentation, such as reducing the expression of HLA-1 by changing the expression level of HLA-1 or affecting the antigen processing mechanism, and how the tumor evade the recognition and killing of NK cells after hiding the antigen presentation defect^52^. Major histocompatibility complex (MHC) is a key molecule for immune cells to recognize antigens, and tumor cells may change the expression of MHC molecules, resulting in cancer immune escape^53^. Tumor cells can also inhibit the function of immune cells through the secretion of certain factors that can lead to immune escape, for example, through the release of PD-L1, which binds to the PD-1 receptor on the immune cells, thereby inhibiting the activity of the immune cells and preventing them from effectively attacking tumor cells^54^. Tumor cells can also achieve immune escape by changing the composition of immune cells in the liver microenvironment. For example, tumor cells can recruit immunosuppressive cells, such as regulatory T cells and myeloid-derived suppressor cells, which can help tumor cells evade immune surveillance^55^. Tumor cells can also alter the liver microenvironment by releasing exosomes. Exosomes are minute vesicles that contain proteins, lipids, glycans, RNA, and DNA from tumor cells^56^. These exosomes can affect cells in the liver microenvironment, including immune cells and stromal cells, which can help tumor cells escape immune surveillance. A study^57^ showed KCs can phagocytose exosomes containing highly expressed miR-135a-5p that enter the liver from the blood circulation, and exosomal miR-135a-5p can activate the large tumor inhibitory kinase 2-Yes-associated protein-matrix metalloproteinase 7 axis to promote the occurrence of liver metastasis. Another study showed that tumor-derived exosome miR-934 induces M2 macrophage polarization through downregulation of PTEN expression and activation of the PI3K/AKT signaling pathway, and polarized M2 macrophages could induce premetastatic ecotope formation and promote liver metastasis through the secretion of CXCL13^58^.

## Chinese medicine application as gastric cancer liver metastasis inhibitors

### Monomer compounds of TCM

The application of Chinese medicines has a long history, and their therapeutic efficacy has been verified by long-term clinical practice. TCM prescriptions are usually composed of multiple herbs, which exert synergistic therapeutic effects through interaction. However, in modern medical research, people have begun to pay attention to the single active ingredient in TCM, namely, TCM monomers, and to study their mechanism of action and efficacy in the treatment of diseases. TCM monomer usually refers to a single compound with specific pharmacological activity extracted from TCM, which is responsible for some pharmacological effects of TCM. The in-depth study of TCM monomer can provide a scientific basis for its application in medicine, health care products, and other fields, which is of great value. At present, TCM monomer compounds have been widely used in the treatment of GCLM. This study summarizes the individual TCM compounds as well as TCM compounds related to GCLM (Table 1).

#### Hydroxysafflor yellow A

Hydroxysafflor yellow A (HSYA) is a monochalcone glycoside isolated and extracted from medicinal saffron. In TCM, saffron is widely used for promoting blood circulation and removing blood stasis, and HSYA is considered one of the most effective water-soluble components of safflower. HSYA can inhibit platelet aggregation and release induced by platelet-activating factor^59^. Modern studies have shown that HSYA has good utility in neuroprotection for the treatment of localized cerebral ischemia^60^. HSYA is also closely related to antioxidation, anti-inflammation, improvement of glucose metabolism and liver function, and coronary artery dilatation^61^. Wang *et al.*^62^ found that HSYA could effectively inhibit oxidative stress-mediated liver injury by increasing antioxidant enzyme activity, stimulating PPARγ activity, reducing cell proliferation, and inhibiting ECM synthesis, thereby alleviating liver fibrosis. Hepatic fibrosis may lead to changes in the microenvironment of the liver, increasing the possibility of GC cell colonization and growth in the liver. Studies also revealed that HSYA inhibits the growth of transplanted human gastric adenocarcinoma BGC-823 tumors in nude mice by observing the pathological changes of tumors and capillarogenesis through light microscopy, and one of the possible mechanisms is through the inhibition of tumor capillarogenesis^63^.

#### Baicalein

BAI is a kind of plant flavonoid extracted from Scutellaria baicalensis, and recent studies have shown that it has a promising role in tumor-related aspects. BAI prevents tumor progression and proliferation by inhibiting tumor cell migration and invasion, and inducing apoptosis of tumor cells^64^. *In vitro* and *in vivo* studies by Dan Qiao *et al.* have shown that BAI interacts with FAK to downregulate AKT/mTOR signaling and inhibit GC cell proliferation and migration. They also investigated the mechanism of BAI inhibiting GC progression by mediating miR-7/FAK/AKT signaling pathway. The results showed that BAI could inhibit FAK expression by upregulating the miR-7 expression or directly targeting FAK 3'UTR to inhibit FAK expression and affects the proliferation, metastasis, and angiogenesis of GC cells through PI3K/AKT signaling pathway^65^,^66^. Xi Yan *et al.*^67^ demonstrated that BAI inhibits the p38 signaling pathway, decreases the expression levels of matrix metalloproteinase (MMP)-2 and -9 in GC cells, and inhibits the invasion and metastasis of GC cells. Other studies have shown that BAI promotes the apoptosis of GC cells by activating the NF-κB signaling pathway and inducing the activation of NLRP3 inflammasome^68^.

#### Curcumin

Curcumin is a polyphenolic compound extracted from the rhizomes of turmeric and mustard in the ginger family. Modern studies have pointed out that curcumin possesses a variety of biological activities, including anti-inflammatory, antioxidation, antitumor, antimicrobial, and hepatoprotective effects^69^. Curcumin also plays a role in GCLM. Studies have found that curcumin can enhance the antitumor immunity of tumor-associated macrophages by shifting M polarization to the M1 phenotype and/or upregulating the expression of M1 markers^70^. Curcumin can inhibit cancer cell proliferation, invasion, metastasis, and angiogenesis through a variety of mechanisms^71^,^72^. A curcumin derivative, CH-5, can inhibit the migration and invasion of human GC cell line HGC-27 by downregulating the expression of matrix metalloproteinase-2 and the activity of collagenase. In addition, CH-5 reduces the viability and induces apoptosis of HGC-27 cells^73^. Zhang *et al.*[74]investigated the functional effects of curcumin on the GC cell line SGC-7901 via Shh and Wnt signaling pathways by using the Transwell invasion assay, immunofluorescence, and flow cytometry techniques. The results showed that curcumin inhibits the expression of Shh, Gli1, and Foxm1 in the Shh signaling pathway and β-catenin in the Wnt signaling pathway, leading to a decrease in the migration and invasion ability of SGC-7901 cells. This study also showed that curcumin induces cytoskeletal remodeling and S-phase cell cycle arrest, and inhibits EMT. However, it has not yet revealed the specific mechanism by which curcumin inhibits the interaction between Gli1 and β-catenin in GC cells, so further studies are needed.

#### Huaier extract

Trametes robiniophila Murr (or Huaier) is a commonly used TCM, and its main active ingredients include polysaccharides, proteins, amino acids, and alkaloids. A number of studies have demonstrated the efficacy of Huaier in inhibiting tumor metastasis^75^. Wang *et al.*^76^ investigated the effect of Huaier on the proliferation and metastasis ability of HGC27, MGC803, and AGS human GC cell lines and the possible mechanism. Huaier n-butanol extract inhibits the invasion and migration of GC cells by reducing the expression of vimentin, an EMT-related marker. Huaier n-butanol extract also inhibits the proliferation, migration, and invasion of GC cells by downregulating c-Myc and Bmi1. Xu *et al.*^77^ also showed that the aqueous extract of Huaier inhibits EMT by targeting Twist, which increases the expression of epithelial marker E-cadherin and decreases the expression of mesenchymal markers N-cadherin and vimentin, and inhibits the invasion and migration ability of GC cells.

#### Berberine

Berberine (BBR), usually extracted from the TCM Rhizoma Coptidis, is a pale yellow crystalline benzylisoquinoline alkaloid that possesses a variety of pharmacological effects, including antioxidant, anti-inflammatory, anticancer, hepatoprotective, hypolipidemic, and hypoglycemic activities^78^. BBR can exhibit good anticancer effects through a variety of mechanisms, such as induction of apoptosis, cell cycle arrest, and inhibition of angiogenesis^79^. According to studies, BBR has a good inhibitory effect on the proliferation, invasion, and migration of GC cells by reducing the expression of MMP-3. Further study found that BBR could reduce the expression of HNF4α, WNT5A, and cytoplasmic β-catenin in cancer tissues, and the regulation of HNF4α-Wnt5a is a crosstalk between AMPK metabolic pathway and WNT signaling pathway. This outcome suggests that the AMPK-HNF4α-WNT5A signaling pathway may be involved in BBR's anti-GC mechanism^80^. BBR inhibits the proliferation, migration, and invasion of GC cells; induces apoptosis; and inhibits tumor growth *in vivo* as evidenced by the use of a nude mice model of xenografts. In addition, the underlying mechanism may be related to the regulation of IL-6/JAK2/STAT3-related signaling pathways by BBR, as investigated by using RNA-Seq, qRT-PCR, WB, and ELISA^81^.

#### Tanshinone IIA

Tanshinone IIA (TSN IIA) is a kind of natural active ingredient of salvia miltiorrhiza, which has been used in TCM to relieve pain, promote blood circulation, and dissolve blood stasis^82^. Modern pharmacological studies have shown that TSN IIA has a wide range of biological activities, including anti-inflammatory, antitumor, antioxidant, and other effects. Reports have indicated that the application of TSN IIA exerts antitumor effects mainly by inhibiting tumor cell proliferation, inducing apoptosis, preventing migration and invasion, and hindering immune escape^83^. Many studies have shown that TSN IIA can inhibit GC proliferation and metastasis. Yu *et al.*^84^ found that the transfection of GC cells with pcDNA-FOXM1 or FOXM1-siRNA or the treatment of GC cells with TSN inhibits cell proliferation and migration. The study also found that overexpression of FOXM1 increases the expression of Ki-67, PCNA, and MMP-2/-9, which reverses the inhibition of TSN IIA-induced proliferation and migration of GC cells, demonstrating the inhibition of GC cell proliferation and migration by downregulation of FOXM1 by TSN IIA. In addition, a study^85^ proved that TSN IIA could limit the proliferation, migration, invasion, and EMT of GC cells by regulating miR-874, and searched for potential miR-874 targeting HMGB2 by bioinformatic methods. Its mechanism may inhibit the proliferation and invasion of GC cells by regulating the miR-874/HMGB2/β-catenin pathway in GC.

#### Resveratrol

Resveratrol (RS) is a polyphenolic compound naturally occurring in grapes, red wine, and other plants, and it has received attention for its possible health benefits, which include anti-inflammatory and antioxidant effect, and inhibition of cancer^86^. Yang *et al.*^87^ found that RS dose-dependently decreases the expression of MALAT7901 in two GC cell lines (BGC1 and SGC823) and inhibits cell viability and proliferation; further studies found that after RS was applied to BGC823 cells transfected with siRNA-1, the expression of waveform protein was significantly reduced (P<1.200), and the expression of E-calmodulin protein was significantly increased, indicating that downregulation of the expression of MALAT1 inhibits EMT in BGC823 cells. RS inhibits cell migration and invasion by inhibiting MALAT1-mediated EMT in GC cell line BGC823. Studies^88^ have shown that the RS can through the MALAT1/miR-383-5 p/DDIT4 axis inhibit GC cell proliferation, migration, and invasion, and induce its apoptosis; RS can reduce the expression of MALAT1 in GC cells and upregulate the expression of miR-383-5p to regulate the expression level of DDIT4 to control the proliferation, migration, invasion, and apoptosis of GC cells.

#### Matrine

Matrine is an alkaloid extracted from the legume plant Matrine. Matrine has a variety of pharmacological effects, including anticancer, anti-inflammatory, antibacterial, and attenuation^89^. Matrine and its derivatives may exert their anticancer effects through a variety of mechanisms, including inhibition of tumor cell proliferation, invasion, and metastasis, and induction of tumor cell apoptosis^90^. Studies have shown that oxymatrine can effectively inhibit the phosphorylation of EGFR (Tyr845), which in turn inhibits the EGFR/Cyclin D1/CDK4/6 pathway, the EGFR/Akt pathway, and the EGFR/MEK-1/ERK1/2/MMP2 pathway to promote apoptosis and inhibit the proliferation and invasion of GC cells. However, this study was not validated from the *in vivo* direction^91^. Vasodilator-stimulated phosphoprotein (VASP) is associated with the proliferation, invasion, and metastasis of malignant tumor cells. Zhang *et al.* showed that matrine causes substantial changes in the secondary structure of VASP protein by CD spectroscopy. Further, laser confocal scanning microscopy showed that matrine affects the subcellular distribution of VASP and the formation of actin stress fibers; cell scratch and adhesion assays, as well as real-time fluorescence quantitative PCR and Western blot, revealed that picloram considerably inhibits the migration and adhesion of BGC823 cells, as well as decreases the expression and phosphorylation of VASP proteins, but it has no remarkable effect on VASP mRNA expression^92^. One study demonstrated a remarkable inhibitory effect of matrine on the migration of SGC7901 cells in 2D and 3D cell migration assays and found that matrine may inhibit the proliferation and metastasis of GC cells by inhibiting the PI3K/Akt/uPA pathway^93^.

#### Celastrol

Celastrol, a natural compound extracted from the TCM Lei Gong Teng, has a variety of biological activities, including antiobesity, antidiabetes, and anticancer properties^94^. In cancer, celastrol shows a substantial broad-spectrum antitumor activity in several kinds of cancer, such as lung cancer, liver cancer, colorectal cancer, and blood system of malignant tumors. In terms of anticancer properties, celastrol exhibits considerable broad-spectrum anticancer activity against a wide range of cancers, such as lung, liver, colorectal, hematological malignancies, and GCs^95^. The action of celastrol is also involved in the regulation of many important signaling pathways, such as phosphatidylinositol 3-kinase (PI3K)/protein kinase B(AKT), and nuclear factor-kB (NF-kB) pathway. Celastrol prevents the proliferation, migration, and invasion of GC cells by downregulating the expression of FOXA1, thereby reducing the transcription of CLDN4, and inhibiting the phosphatidylinositol 3-kinase (PI3K)/protein kinase B (AKT) pathway^96^. In addition, studies have shown that celastrol can remarkably inhibit cell proliferation, migration, and invasion, and induce apoptosis and G2/M cell cycle arrest in human GC cell line MKN45. Further research reveals it may inhibit the proliferation, migration, and invasion in MKN45 cells by downregulating the expression of microRNA-21 (miR-21), and then inactivating the PTEN/PI3K/AKT and NF-κB signaling pathways^97^.

#### Others

Zhang *et al.*^98^ reported that acacetin could inhibit the liver metastasis of GC cells. Experiments showed that acacetin inhibits the invasion, metastasis, and angiogenesis of GC cells by downregulating the expression of N-cadherin, upregulating the expression of E-cadherin, and controlling the expression of MMPs. Further studies also found that acacetin inhibits the invasion, metastasis, and EMT of GC cells by inhibiting the PI3K/Akt/Snail signaling pathway. Zhu *et al.*^99^ showed that Astragaloside IV (AS-IV) affects GC invasion and metastasis by inhibiting EMT, and the possible mechanism is that AS-IV could reverse the activation of PI3K/Akt/NF-κB induced by TGF-β1 and inhibit the conversion of E-cadherin to N-cadherin and the expression of EMT-related genes. Zhou *et al.*^100^ found Vitexin has a substantial inhibitory effect on GCLM. Vitexin inhibits the migration, invasion, and EMT of SGC-7901 and AGS cells in a dose-dependent manner. Vitexin causes the phosphorylation of PI3K, AKT, and mTOR and decreases the expression of HIF-1α, implying that Vitexin inhibits the activation of the PI3K/AKT/mTOR/HIF-1α pathway and thus prevents the EMT of GC cells. This study further indicated that overexpression of HMGB1 promotes the activation of PI3K/AKT/mTOR/HIF-1α pathway and vitexin could inhibit the overexpression of HMGB1. In addition, betulinic acid is a good drug for targeting cancer stemness. Chen *et al.* demonstrated that betulinic acid effectively inhibits GRP78-mediated TGF-β secretion in a dose-dependent manner, which further inhibits macrophage polarization and IL-6 secretion to inhibit GC cell stemness^101^.

### Compound traditional Chinese medicine

Compound Chinese medicine or TCM compound prescription refers to the use of two or more Chinese medicines in combination to treat diseases. TCM compound is the core component of TCM, which shows unique therapeutic effects through the comprehensive action of multiple components, pathways, and targets. Modern research is also striving to explore the active components and mechanisms of action of TCM compound to improve the utilization of these traditional medicine resources and the clinical therapeutic effects.

#### Si Jun Zi Decoction

Sijunzi Decoction is a classic prescription in TCM, which was first seen in the Taiping Huimin Mixture Bureau. Sijunzi Decoction consists of ginseng, licorice, atractylodes, and poria, and has the overall effect of tonifying the spleen and benefiting the stomach. Components such as ketone, ginsenoside, and atractylodes help Sijunzi decoction achieve antitumor effects. Ding *et al.* conducted data mining and statistical analysis of the mechanism of Sijunzi Decoction in the treatment of GC based on network pharmacology, and the study showed that Sijunzi Decoction inhibits angiogenesis by downregulating the expression of VEGFA, iNOS, and COX-2, and induces cell apoptosis by upregulating the ratio of Bax/Bcl2 to regulate the PI3K/AKT pathway, so the further development of GC is inhibited^102^.

#### Gancao Xiexin Decoction

Gancao Xiexin Decoction originates from the “Shang Han Za Bing Lun” (Treatise on Cold Damage and Miscellaneous Diseases), and its composition includes licorice, rhubarb, coptis chinensis, scutellaria baicalensis, and alisma. In modern clinical practice of TCM, this prescription is commonly used with modifications to treat digestive system diseases such as acute and chronic gastritis and gastric ulcers. Studies^103^ have shown that Gancao Xiexin Decoction can reduce cell migration and invasion, block the cell cycle, inhibit the proliferation of GC cells, and increase the number of apoptosis of GC cells. Yang *et al.* first used network pharmacology as the preliminary basis and then cell experiments demonstrated that the mechanism of Gancaoxixin Decoction inhibits the proliferation and migration, induces cell apoptosis, and the blocks the cell cycle of GC by inhibiting the JAK2/STAT3 signaling pathway.

#### Lizard Stomach Comfort Formula

Lizard Stomach Comfort Formula is mainly composed of Eremias multipunctatus, Radix pseudostellariae, Astragalus, Dendrobium, Prunus mume, fried white paeony, snakeberry, Citrus aurantium, Scutellaria barbata, and Panax notoginseng powder. This prescription takes Eremias multipunctatus as the main drug, which has the functions of deciphering and dispersing nodules, promoting blood circulation, and removing blood stasis, as well as resisting cancer. Snakeberry and Scutellaria barbata assist Eremias multipunctatus in clearing heat and eliminating tumors. Radix pseudostellariae and Astragalus can benefit qi and nourish blood, and improve the microenvironment of ischemia and hypoxia. Studies have shown that the Lizard Stomach Comfort Formula has a good effect on the treatment of GCLM. It can promote the apoptosis of GC cells and inhibit GCLM by downregulating the expression of HIF-1α, PI3K, and p-AKT proteins in the PI3K/AKT signaling pathway mediated by HIF-1α^104^.

## Summary and Prospects

GCLM is an important sign of the deterioration of GC, which is an important research field in the prevention and treatment of tumor metastasis, and an important problem to be solved urgently. The GCLM mechanism is complex, involving the shedding of tumor cells, invasion, angiogenesis, extracellular matrix degradation, and transformation of epithelial-mesenchymal multiple links. TCM is safe and effective and has few side effects. Its mechanism of treating GCLM is to regulate the internal balance of the body and inhibit the growth and metastasis of tumors through the multicomponent, multitarget action of TCM. As mentioned above, Huaier n-butanol extract and the aqueous extract of Huaier can reduce the occurrence of GCLM by inhibiting EMT, proliferation, and other effects. Many TCM compound extracts, TCM active ingredients, and monomer compounds can inhibit the growth and metastasis of GC. However, compared with TCM and TCM monomers, studies on TCM compounds are fewer, and therapeutic agents that affect the GCLM due to mechanisms such as tumor cell stemness, ECM degradation, and circulating tumor cells are lacking, so much research is still needed on them. At present, the combination of TCM and modern medicine provides new ideas and methods for the treatment of GCLM. Although the treatment of TCM has some advantages, it has certain limitations. The composition of TCM is complex, and differences may exist between diverse varieties and qualities of TCM, so it lacks persuasive power and brings uncertainty to clinical application. With the deepening of research and the application of modern science and technology, the efficacy and mechanism of TCM will be more clearly defined, and its role in the treatment of GC will be better utilized.

## Figures and Tables

**Table 1:** Summary of the effects of different Chinese Medicine Monomers or Herbal Formula of TCMs on GCLM

No.	Chinese Medicine Monomers/Herbal Formula	Source	Functions/Target	Pathway	References
1	Hydroxysafflor yellow A	Carthamus tinctorius	Alleviate liver fibrosis	—	62
			Inhibite tumor capillarogenesis	—	63
2	Baicalein	Scutellaria baicalensis	Inhibit proliferation and angiogenesi	miR-7/FAK/AKT pathway	66
			Inhibition of invasion and metastasis	P38 pathway	67
			Activation of NLRP3 induced apoptosis	NF-κB pathway	68
3	Curcumin	Turmeric	Induce apoptosis	—	73
			Inhibite EMT	Shh pathway、Wnt pathway	74
4	Huaier Extract	Trametes robiniophila Murr	Inhibit proliferation, invasion and migration	—	76 77
5	Berberine	Coptis chinensis	Inhibit proliferation, migration and invasion	AMPK-HNF4α-WNT5A pathway	80
			Inhibit proliferation, migration and invasion	IL-6/JAK2/STAT3 pathway	81
6	Tanshinone IIA	Salvia miltiorrhiza	FOXM1	—	84
			Inhibite of invasion and metastasis	miR-874/HMGB2/β-catenin pathway	85
7	Resveratrol	Grape, red wine etc.	Inhibite EMT	MALAT1	87
			Induced apoptosis	MALAT1/miR-383-5p/DDIT4 pathway	88
8	Matrine	Sophora flavescens	Inhibit proliferation and metastasis	PI3K/Akt/uPA pathway	93
9	Celastrol	Tripterygium wilfordii	Inhibit proliferation, migration and invasion	PI3K/AKT pathway	96
			Inhibit proliferation, migration and invasion	NF- κB pathway	97
10	Acacetin	Robinia pseudoacacia	Inhibition of invasion and metastasis and EMT	PI3K/Akt/Snail pathway	98
11	Astragaloside IV	Astragalus membranaceus	Inhibition of EMT	PI3K/Akt/NF-κB pathway	99
12	Vitexin	Vitex negundo	Inhibition of invasion and metastasis and EMT	PI3K/AKT/mTOR/HIF-1αpathway	100
13	Betulinic acid	White birch	Suppressing stemness in gastric cancer cells	GRP78-TGF-β1	101
14	Si Jun Zi Decoction	—	Inhibit angiogenesis	PI3K/AKT pathway	102
15	Gancao Xiexin Decoction	—	Inhibition of invasion and metastasis	JAK2/STAT3 pathway	103
16	Lizard Stomach Comfort Formula	—	Improve microenvironment and promote apoptosis	PI3K/AKT pathway	104
